# Activation and regulation of the granulation tissue derived cells with stemness-related properties

**DOI:** 10.1186/s13287-015-0070-9

**Published:** 2015-04-29

**Authors:** Zelin Chen, Tingyu Dai, Xia Chen, Li Tan, Chunmeng Shi

**Affiliations:** Institute of Combined Injury, State Key Laboratory of Trauma, Burns and Combined Injury, Chongqing Engineering Research Center for Nanomedicine, College of Preventive Medicine, Third Military Medical University, Chongqing, 400038 China

## Abstract

**Introduction:**

Skin as the largest and easily accessible organ of the body represents an abundant source of adult stem cells. Among them, dermal stem cells hold great promise in tissue repair and the skin granulation tissue has been recently proposed as a promising source of dermal stem cells, but their biological characteristics have not been well investigated.

**Methods:**

The 5-bromo-2′-deoxyuridine (BrdU) lineage tracing approach was employed to chase dermal stem cells in vivo. Granulation tissue derived cells (GTCs) were isolated and their in vitro proliferation, self-renewing, migration, and multi-differentiation capabilities were assessed. Combined radiation and skin wound model was used to investigate the therapeutic effects of GTCs. MicroRNA-21 (miR-21) antagomir was used to antagonize miR-21 expression. Reactive oxygen species (ROS) were scavenged by N-acetyl cysteine (NAC).

**Results:**

The quiescent dermal stem/progenitor cells were activated to proliferate upon injury and enriched in granulation tissues. GTCs exhibited enhanced proliferation, colony formation and multi-differentiation capacities. Topical transplantation of GTCs into the combined radiation and skin wound mice accelerated wound healing and reduced tissue fibrosis. Blockade of the miR-21 expression in GTCs inhibited cell migration and differentiation, but promoted cell proliferation and self-renewing at least partially via a ROS dependent pathway.

**Conclusions:**

The granulation tissue may represent an alternative adult stem cell source in tissue replacement therapy and miR-21 mediated ROS generation negatively regulates the stemness-related properties of granulation tissue derived cells.

**Electronic supplementary material:**

The online version of this article (doi:10.1186/s13287-015-0070-9) contains supplementary material, which is available to authorized users.

## Introduction

Stem cell-based therapy has aroused great promise in regenerative medicine and an adult stem cell source is a key resource for clinical application. Skin, the largest organ of the body, is emerging as a reservoir for adult stem cells. Stem cells have been proven to exist in the epithelial layer of the skin including epidermis [1-4] and appendages [5,6]. Recently, dermis – the stromal part of the skin – has been demonstrated as a promising source of stem cell populations with high self-renewing and multilineage differentiation capacities [7]. However, dermis-derived stem cells in normal adults are relatively rare [8]. Very recently, granulation tissue has been proposed as a potential source of dermal stem cells, and stem cells derived from the granulation tissue could improve the recovery of kidney and liver injury [9,10]. However, their biological characteristics were poorly understood. It has been increasingly established that stem cells play an important role in wound healing and granulation tissue formation warrants proliferation and differentiation of dermal stem cells. In this study, we identified that dermal stem/progenitor cells were activated after wounding by the 5-bromo-2′-deoxyuridine (BrdU) lineage tracing approach. Granulation tissue-derived cells (GTCs) were successfully isolated and exhibited enhanced proliferation, colony formation, and multidifferentiation capabilities compared with nonwounded adult dermal cells. Topical transplantation of the GTCs accelerated wound healing and reduced tissue fibrosis in mice with combined radiation and skin wound injury. Furthermore, microRNA (miR)-21 and reactive oxygen species (ROS) were significantly upregulated in these cells, and miR-21 was shown to promote cell migration and differentiation, but inhibit cell proliferation and self-renewing at least partially via a ROS-dependent pathway.

## Methods

### Animals

C57/BL mice were obtained from the Center of Experimental Animal at the Third Military Medicine University (TMMU, Chongqing, China). The experiments were conducted in accordance with the Guidelines for the Care and Use of Laboratory Animals of the TMMU, and all procedures were approved by the Animal Care and Use Committee of the TMMU.

### Skin wound model

The skin wound model was performed as described previously [11]. In brief, mice were anesthetized with 1% pentobarbital (30 mg/kg), and the back hair was shaved. Circular, full-thickness skin excisions of 10 mm in diameter were surgically made in the middle back of each animal.

### Cell isolation and culture

For neonatal and adult dermal cell isolation, dorsal skin was carefully dissected free of other tissue, cut into 1 to 2 mm^3^ pieces, and washed with phosphate-buffered saline (PBS) three times. After being digested with 0.25% trypsin–ethylenediamine tetraacetic acid (HyClone, Logan, UT, USA) at 4°C overnight, the epidermis was removed, and the remaining dermal parts were further digested with 0.25% collagenase I (Worthington, Biochemical Corporation, Lakewood, NJ, USA), and shaken at 37°C for another 2 hours. The digested cells were then passed through a 75 μm cell strainer (Sangon Biotech, Shanghai, China), centrifuged, and resuspended in Iscove's Modified Dulbecco's Media (HyClone), supplemented with 10% fetal bovine serum (Gibco, Grand Island, NY, USA), 100 U/ml penicillin and 0.1 mg/ml streptomycin (all from Beyotime, Shanghai, China). Cells were seeded in a tissue culture flask at 1 × 10^3^ cells/cm^2^, and maintained at 37°C with 5% carbon dioxide. After 24 hours, the plates were washed with PBS to remove residual nonadherent cells. The remaining adherent cells were subcultured after exposure to 0.25% trypsin–ethylenediamine tetraacetic acid (HyClone) every 3 days. The cells of passage 2 or 3 were used for further experiments. For GTC isolation, the granulation tissues at 7 days after wounding were excised and cut into 1 to 2 mm^3^ pieces, washed with PBS three times, then digested with 0.25% collagenase I (Worthington, Biochemical Corporation), and shaken at 37°C for 2 hours. Subsequently, they were processed in the same way as described above to harvest the adherent cells. To antagonize the miR-21 expression, cells were pretreated with miR-21 antagomir (50 nM; Ribobio Co., Guangzhou, China) for 48 hours. For scavenging the intracellular ROS, *N*-acetylcysteine (NAC, 5 mM; Sigma, St. Louis, MO, USA) was added into the culture medium or induced medium.

### 5-Bromo-2′-deoxyuridine pulse chase

BrdU (100 mg/kg; Sigma), a synthetic nucleoside analog of thymidine, is able to be incorporated into dividing cells. To measure the transient proliferation of the wound tissues, BrdU was injected intraperitoneally 2 hours prior to tissue harvest, and the nonwounded control skin or wounded tissues (*n* = 3 mice per time point) were excised and fixed in 4% paraformaldehyde at 1, 3, 5, 7, 9, 12, and 15 days after wounding. For label-retaining cell (LRC) detection, BrdU was injected intraperitoneally six times with 12-hour intervals into wounded mice (long-term BrdU labeling group) or into the nonwounded mice (control group). To measure the time of the transient-amplifying cells retaining the BrdU label, BrdU was injected intraperitoneally once at 60 hours after wounding (a single BrdU labeling group). On post-wound days 3, 7, 9, 12, and 15, the wound tissues of the three groups (*n* = 3 mice per time point for each group) were excised and fixed in 4% paraformaldehyde. The signal of BrdU was detected by immunohistochemistry. For *in vitro* LRC detection, BrdU was injected intraperitoneally six times with 12-hour intervals immediately after wounding (*n* = 6 mice) or into the nonwounded mice (*n* = 6 mice). The nonwounded dermal cells or GTCs at 7 days after wounding were isolated and the adherent cells were reseeded in a six-well plate at a density of 1 × 10^3^ /well (*n* = 6 wells per group) and cultured for another 8 days. Subsequently, the cells were fixed in 4% paraformaldehyde, and the colonies were counted. The signal of BrdU was detected by immunofluorescence.

### Combined radiation and skin wound model and GTC-based treatment

The total body radiation injury (6 Gy) was made by a ^60^Co γ-ray source. The absorption rate was 31.02 to 31.98 cGy/minute. Mice were anesthetized with 1% pentobarbital (30 mg/kg) after radiation, and the back hair was shaved. Circular, full-thickness skin excisions of 8 mm in diameter were surgically made in the middle back of each animal. For treatment, 1 × 10^6^ GTCs suspended in 200 μl PBS were intradermally injected around each wound margin. PBS (200 μl) without cells was used as the control. At indicated time points, wounds (*n* = 6 in the transplantation group or control group) were photographed and quantified by Image-J software (Rasband, W.S., ImageJ, U.S. National Institutes of Health, Bethesda, Maryland, USA). After the wound healed, the wound repair bed and surrounding tissues were obtained. Paraffin sections were stained by Sirius Red or immunostained with α-smooth muscle actin antibody. The fibrotic tissue depths were quantified in serial sections in the center of wounds treated with GTCs or PBS.

### Institutional approval

All procedures on these animals were approved by the Animal Care and Use Committee of the TMMU. All animal experimentation methodology was carried out in accordance with the Guidelines for the Care and Use of Laboratory Animals of the TMMU and the Guidelines on Care and Use of Laboratory Animals issued by the Chinese Council on Animal Research and the Guidelines of Animal Care.

### Tissue staining

Immunohistochemistry was performed as described previously [11]. The rabbit anti-BrdU (1:200; Rockland, Philadelphia, Pennsylvania, USA) was used and detected in 3,3′-diaminobenzidine. Slides were counterstained with hematoxylin. The percentage of BrdU-positive cells was calculated by counting the total number of basal cells of granulation tissues and cells expressing nuclear BrdU stain.

Immunofluorescence was also performed as described previously [12]. The rabbit anti-BrdU (1:200; Rockland) or mouse monoclonal anti-α-smooth muscle actin antibody (1:300; Sigma) was used and detected with secondary donkey anti-mouse or rabbit IgG-Cy3 antibody (1:200; Beyotime). Cells were counterstained with the nuclear dye 4′,6-diamidino-2-phenylindole (Beyotime) and examined with a fluorescence microscope.

Sirius red staining was performed as described previously [11]. In brief, paraffin-embedded sections were dehydrated and stained in Sirius red solution for 1 hour, then mounted with Poly-Mount Xylene. Polaroid lens were used for images taken under a microscope.

### Quantitative real-time PCR analysis of miR-21 expression

Total RNA was extracted using RNAiso Plus (TaKaRa, Kyoto, Japan). For the miR-21 expression detection, stem-loop RT-PCR was performed as described previously [13]. Quantitative PCR was carried out using a SYBR® Premix Ex Taq™ II (Tli RNaseH Plus) real-time PCR kit (TaKaRa) according to the manufacturer’s protocol. Relative expression was normalized to the expression of U6 small RNA. The primers for miR-21 and U6 were purchased from Ribobio Co. (Guangzhou, China). Three independent experiments were performed. In each experiment, triplicate procedures were performed for each group.

### Measuring the endogenous ROS level

The ROS-sensitive dye 2′,7′-dichlorofluorescin diacetate (10 mM; Beyotime) was used to measure the endogenous cellular ROS level according to the manufacturer’s protocol. In brief, the primary cells were incubated with 2′,7′-dichlorofluorescin diacetate for 20 minutes at 37°C with reverse shaking every 3 to 5 minutes. The levels of intracellular ROS were analyzed by measuring the mean fluorescence intensity of 2′,7′-dichlorofluorescein using a flow cytometer as described previously [14]. The data were analyzed by FlowJo 7.6.1 software (Tree-Star, Ashland, OR, USA). Three independent experiments were performed. In each experiment, triplicate procedures were performed for each group.

### Cell proliferation assay

Cells were seeded into 96-well plates at 1 × 10^3^ cells/well and a volume of 100 μl. A CCK-8 Kit (Dojindo, Kumamoto, Japan) was used to measure the cell proliferation at 0, 2, 4, 6, and 8 days after seeding according to the manufacturer’s recommendations. After incubation for 2 hours, the absorbance was measured at 450 nm by a Model 680 Microplate Reader (Bio-Rad, Hercules, CA, USA). Three independent experiments were performed. Each experiment was performed in sextuplicate for each group.

### Colony-forming unit fibroblast assay

Cells (1 × 10^3^) were seeded into each well of a six-well plate and incubated for 12 days in a humidified atmosphere (37°C, 5% carbon dioxide). Culture medium was changed every 3 days. Subsequently, cultures were stained with a Giemsa kit (Nanjing Jiancheng Bioengineering Institute, Nanjing, China) according to the manufacturer’s recommendations. Colonies with 50 or more cells in each well of the six-well plate (*n* = 6 wells for each group) were counted in the microscope. Twenty colonies in each group were randomly selected for mean colony sizes and size distribution analysis. The size of the colonies was measured by Image-J software [15]. Three independent experiments were performed.

### Scratch wound closure assay

We used the scratch wound assay to measure the migration of the dermal derived cells as described previously [16]. In brief, 5 × 10^5^ cells/well were seeded into six-well plates and cultured for 100% confluence, and then maintained for another day in normal culture medium. A straight line through the cell sheet was made by a p200 pipette tip. Each well was washed with PBS to remove the cells debris and replaced with low serum (2%) culture medium for the cell proliferation inhibition. Two markings created by a razor blade on the outer bottom of each well were used as reference points close to the scratch. Images were captured at the reference points every 12 hours until the wounds were closed (100% confluent) with a microscope (Olympus, Kyoto, Japan). The relative migration distances were measured by Image-J software. Three independent experiments were performed. In each experiment, triplicate was performed for each group.

### Differentiation assay

For adipogenic differentiation, cells were seeded into six-well plates with mouse mesenchymal stem cell adipogenic differentiation medium (Cyagen Biosciences, Guangzhou, China) according to the supplier’s instructions. After 3 weeks of differentiation, cells were washed with PBS and then fixed in 10% formalin for 20 minutes and stained with Oil-Red O (USA) solution for image acquisition. The intracellular Oil-Red O was extracted with isopropyl alcohol for quantification. The absorbance of the extracted Oil-Red O was measured at 490 nm using a Model 680 Microplate Reader (Bio-Rad). For osteogenic differentiation, cells were seeded into 24-well plates with mouse mesenchymal stem cell osteogenic differentiation medium (Cyagen Biosciences) according to the supplier’s instructions. After 3 weeks of differentiation, cells were washed with PBS and then fixed in 10% formalin for 20 minutes and stained with Alizarin Red (Sigma) solution for image acquisition. An osteogenesis assay kit (Millipore, Billerica, MA, USA) was applied to quantify the Alizarin Red according to supplier’s instructions.

### Statistical analysis

Statistical analyses were performed using the SPSS 13.0 package (SPSS Inc., Chicago, IL, USA). Data were expressed as mean ± standard deviation. An independent-samples *t* test was used to determine the significant differences between two groups. Comparisons of multiple groups were performed with one-way analysis of variance with corrections for multiple comparisons. *P* <0.05 was considered statistically significant.

## Results

### Activation of dermal stem/progenitor cell proliferation after wounding

**Figure 1:**
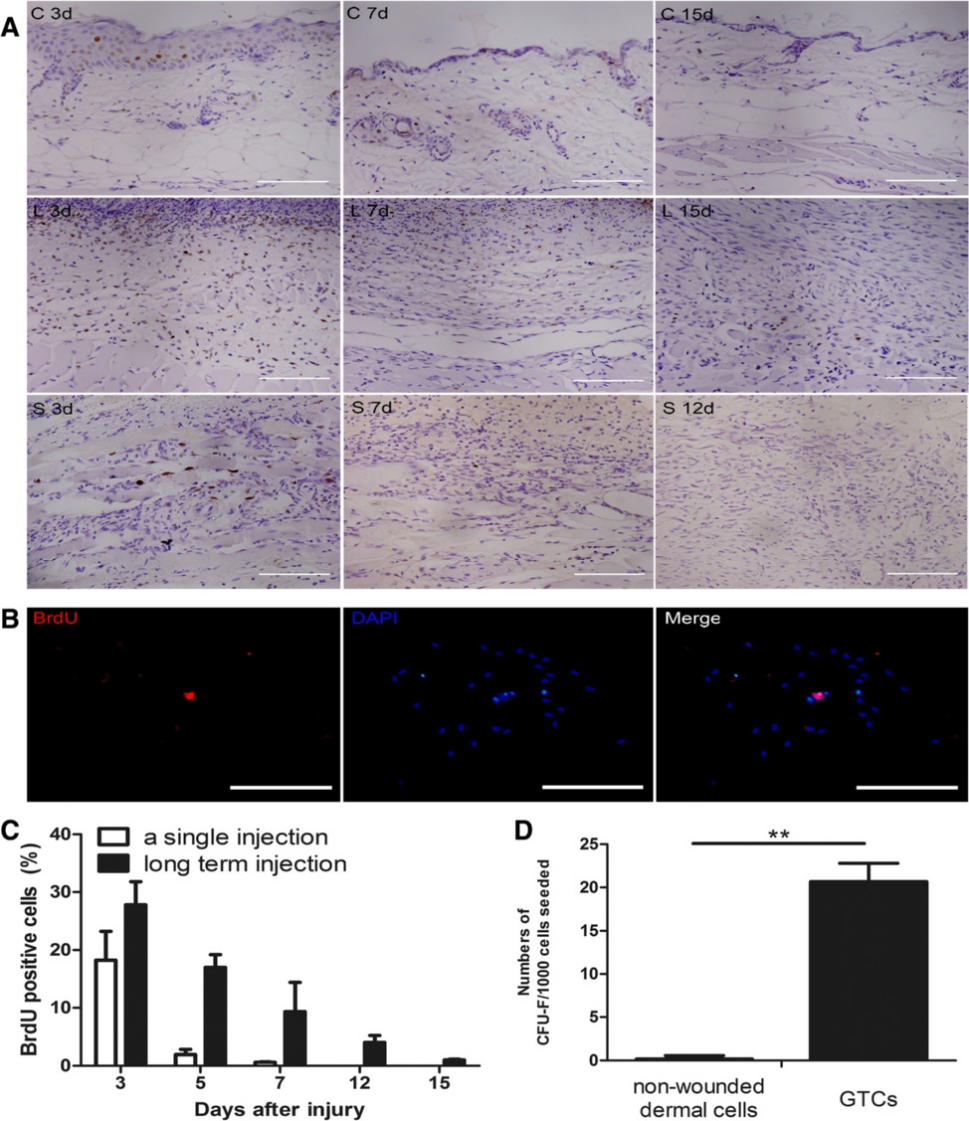
Activation of dermal stem/progenitor cell proliferation after wounding. **(A)** At 3, 5, 7, 12, and 15 days after wounding, 5-bromo-2′-deoxyuridine (BrdU) labels in the normal skin tissues or wounded tissues were detected by immunohistochemistry. The percentage of BrdU-positive cells was also calculated, presented as mean ± standard deviation **(C)**. C, control group (*n* = 3 per time point); d, days after wounding; L, long-term BrdU labeling group (*n* = 3 per time point); S, a single BrdU labeling group (*n* = 3 per time point). Scale bar = 100 μm. **(B)** BrdU (100 mg/kg) was injected intraperitoneally for three consecutive days (once per 12 hours) in nonwounded or wounded mice. Nonwounded dermal cells or granulation tissue-derived cells (GTCs) at 7 days post wounding were isolated and adherent cells were harvested for colony-forming assay. BrdU labels in the colonies were detected by immunofluorescence. Scale bar = 500 μm. **(D)** Quantification of the colonies. CFU, colony-forming unit. **P < 0.01.

It has been long accepted that LRCs represent the stem cell populations in the skin [17], brain [18], bladder [19], and so forth. Thus, we chose the BrdU pulse chase approach to identify the activated dermal stem/progenitor cells *in vivo*. We treated the wounded mice with BrdU for 3 days after wounding. As shown in Figure 1A,C, BrdU-positive cells decreased sharply during the healing process, but were still found in the lower dermal part of the healed wound bed (about 1.03 ± 0.11%). However, no transient-amplifying cells after a single BrdU injection were detected 12 days after wounding (Figure 1A,C). In the nonwounded dermis, no BrdU-positive LRCs or transient-amplifying cells were found (Figure 1A). In addition, we stained the well-known stem cell markers (Sox2, Oct4, CD133) in the granulation tissues at 3, 5, 7, 9, 12, and 15 days after wounding, but no positive stained cells were found (data not shown). Further, we isolated the BrdU-labeled GTCs to test the LRCs *in vitro*. After 8 days of culture, the BrdU-labeled GTCs could form colonies (20.66 ± 2.13; Figure 1D), and BrdU-positive LRCs were also found in some colonies (Figure 1B), while nonwounded dermal cells failed to form colonies (Figure 1D). These results suggested that resident dermal stem/progenitor cells were quiescent in nonwounded dermis, and were activated after wounding.

### Stemness-related properties of granulation tissue-derived cells

**Figure 2:**
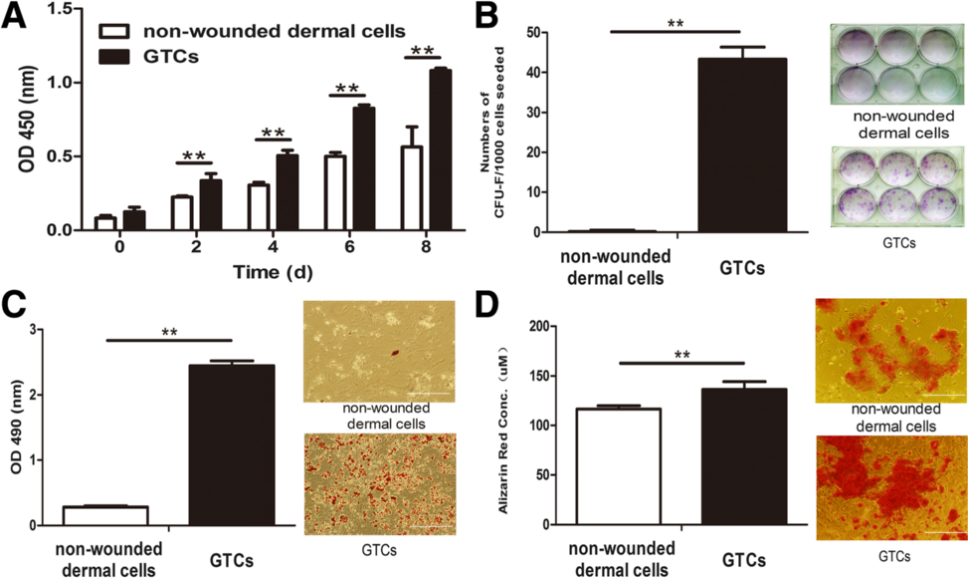
Stemness-related properties of granulation tissue-derived cells. Passage 2 granulation tissue-derived cells (GTCs) or nonwounded dermal cells were used for experiments. **(A)** The proliferation ability of GTCs and nonwounded dermal cells was detected every 2 days after seeding. **(B)** Colony-forming assay of GTCs and nonwounded dermal cells. Results expressed as mean ± standard deviation (*n* = 6 wells per group). **(C)** Adipogenic differentiation was induced in mouse mesenchymal stem cell adipogenic differentiation medium. After 22 days, cells were fixed and stained by Oil-Red O. The Oil-Red O staining was also quantified. **(D)** Osteogenic differentiation was performed in mouse mesenchymal stem cell osteogenic differentiation medium. After 21 days, cells were fixed and stained by Alizarin Red. The Alizarin Red staining was also quantified. d, days; OD, optical density. **P <0.01. Scale bar = 500 μm.

Before isolating the GTCs, we measured the transient proliferation of the granulation tissues by the 2 hours BrdU pulse approach. The proliferation response of dermal tissues initiated at 3 days after wounding reached a peak at 7 days after wounding, and diminished at 15 days (Additional file 1). Thus, we chose to isolate GTCs at 7 days after wounding. We then tested their stemness-related properties. GTCs exhibited much higher proliferation capacity than nonwounded dermal cells (Figure 2A). GTCs formed 43.33 ± 3.01 colonies after 12 days culture, while nonwounded cells failed to form colonies (Figure 2B). In adipogenic differentiation assay, GTCs produced more adipogenic phenotypic cells stained by Oil-Red O, while nonwounded dermal cells exhibited fewer phenotypic changes. Further, the optical density level of Oil-Red O in induced GTCs (2.44 ± 0.07) was significantly higher than in nonwounded dermal cells (0.28 ± 0.02; Figure 2C). In osteogenic differentiation assay, GTCs produced more calcium deposition with strong staining of Alizarin Red (136.43 ± 7.82) than nonwounded dermal cells (116.6 ± 3.36; Figure 2D). These results suggested that the proportion of adult dermal stem cells in nonwounded dermis was very low. After wounding, these cells were activated, enriched in granulation tissues, and exhibited enhanced stemness-related properties.

### Therapeutic effects of GTCs on combined radiation and skin wound injury

**Figure 3:**
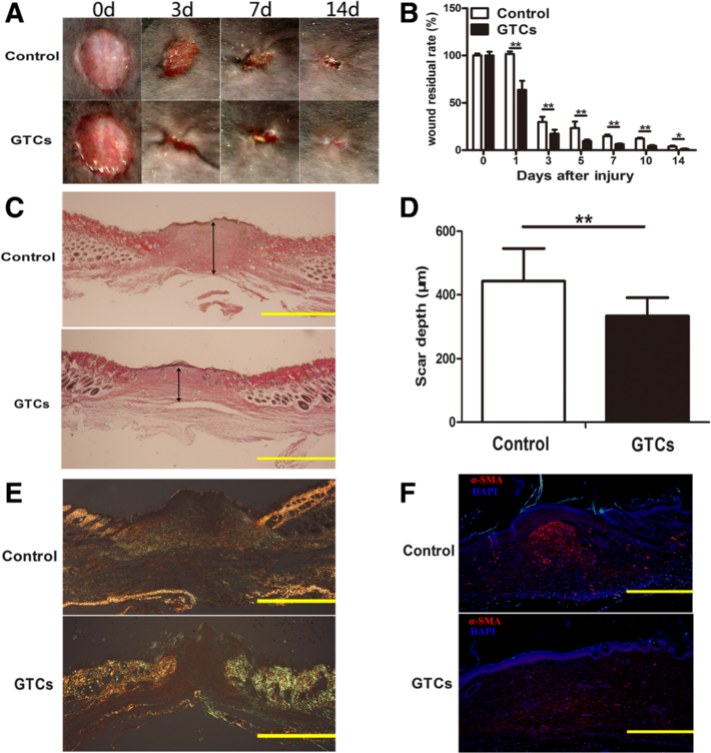
Therapeutic effects of granulation tissue-derived cells on combined radiation and skin wound injury. Granulation tissue-derived cells (GTCs), 1 × 10^6^ per mouse in 0.2 ml phosphate-buffered saline (PBS), were injected around the skin wound margins post 6 Gy total body radiation combined skin wound injury. PBS (0.2 ml per mouse) served as control. **(A)** Representative wounds injected with PBS (control) or GTCs. **(B)** Wound residual rates of the GTC-treated group and the control group are presented as mean ± standard deviation (*n* = 6 mice per group). **(C)** The fibrotic tissue depths were quantified in serial sections in the center of day 15 wounds treated with PBS or GTCs. **(D)** Results of the fibrotic tissue depths presented as mean ± standard deviation (*n* = 6). **(E)** Representative photomicrographs of Sirius Red-stained sections from day 15 wounds injected with PBS or GTCs. **(F)** Immunostaining for the classical myofibroblast-specific marker alpha smooth muscle actin (α-SMA) of wound sections derived from day 15 wounds injected with PBS or GTCs. Scale bars = 500 μm. d, days; DAPI, 4′,6-diamidino-2-phenylindole. **P <0.01, *P <0.05.

It has been well documented that ionizing radiation can delay the wound healing process [20]. In order to test the therapeutic potential of GTCs, a combined radiation and skin wound injury mouse model was further employed in this study. Results showed that GTC transplantation significantly accelerated wound closure (Figure 3A,B). In addition, quantification of fibrotic tissue depth from day 15 (the time of wounds healed) wounds confirmed a significant reduction in fibrotic tissue in GTC-treated wounds (333.34 ± 57.93 μm) compared with that in PBS-treated wounds (443.21 ± 52.49 μm; Figure 3C,D). Moreover, Sirius Red staining for fibrillar collagens revealed less collagen deposition in GTC-treated wounds (Figure 3E). Further results also showed that the myofibroblast marker α-smooth muscle actin-positive cells were lower in the day 15 wounds of GTC-treated wounds (Figure 3F). These data showed that GTC transplantation could promote wound healing and reduce tissue fibrosis in the combined radiation and skin wound injury, suggesting the therapeutic implications of GTCs in tissue replacement therapy.

### miR-21 is a negative regulator of dermal-derived cells for stemness-related properties

**Figure 4:**
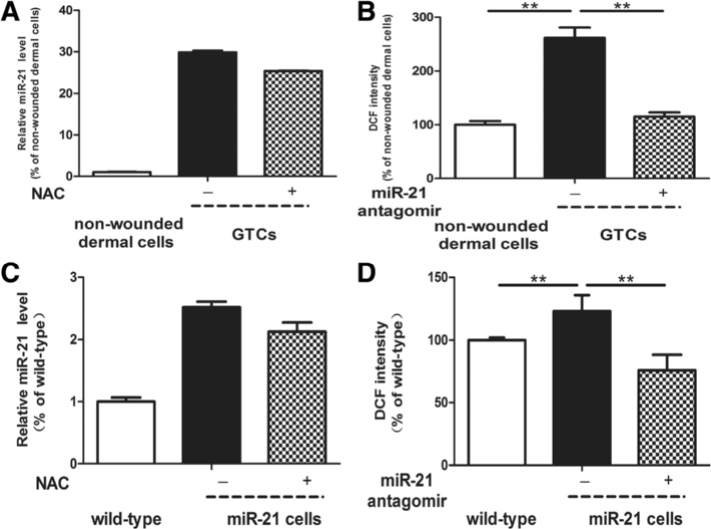
miR-21 regulates the reactive oxygen species level of dermal-derived cells. **(A)** Relative microRNA (miR)-21 levels of primary nonwounded dermal cells, granulation tissue-derived cells (GTCs), and GTCs pretreated with *N*-acetylcysteine (NAC; 5 mM) for 48 hours were tested by quantitative real-time PCR. **(B)** Relative reactive oxygen species (ROS) levels of primary nonwounded dermal cells, GTCs, and GTCs pretreated with miR-21 antagomir (50 nM) for 48 hours were detected by the ROS-sensitive dye 2′,7′-dichlorofluorescin diacetate (DCFH-DA; 10 mM). **(C)** Relative miR-21 levels of primary wild-type neonatal dermal cells, miR-21 knock-in neonatal dermal cells, and miR-21 knock-in neonatal dermal cells pretreated with NAC (5 mM) for 48 hours were detected by quantitative real-time PCR. **(D)** Relative ROS levels of primary wild-type neonatal dermal cells, miR-21 knock-in neonatal dermal cells, and miR-21 knock-in neonatal dermal cells pretreated with miR-21 antagomir (50 nM) for 48 hours were measured by DCFH-DA (10 mM). All results presented as mean ± standard deviation. d, days; DCF, 2′,7′-dichlorofluorescin; h, hours; miR-21 cells, miR-21 knock-in neonatal dermal cells; wild-type, wild-type neonatal dermal cells. **P < 0.01.

**Figure 5:**
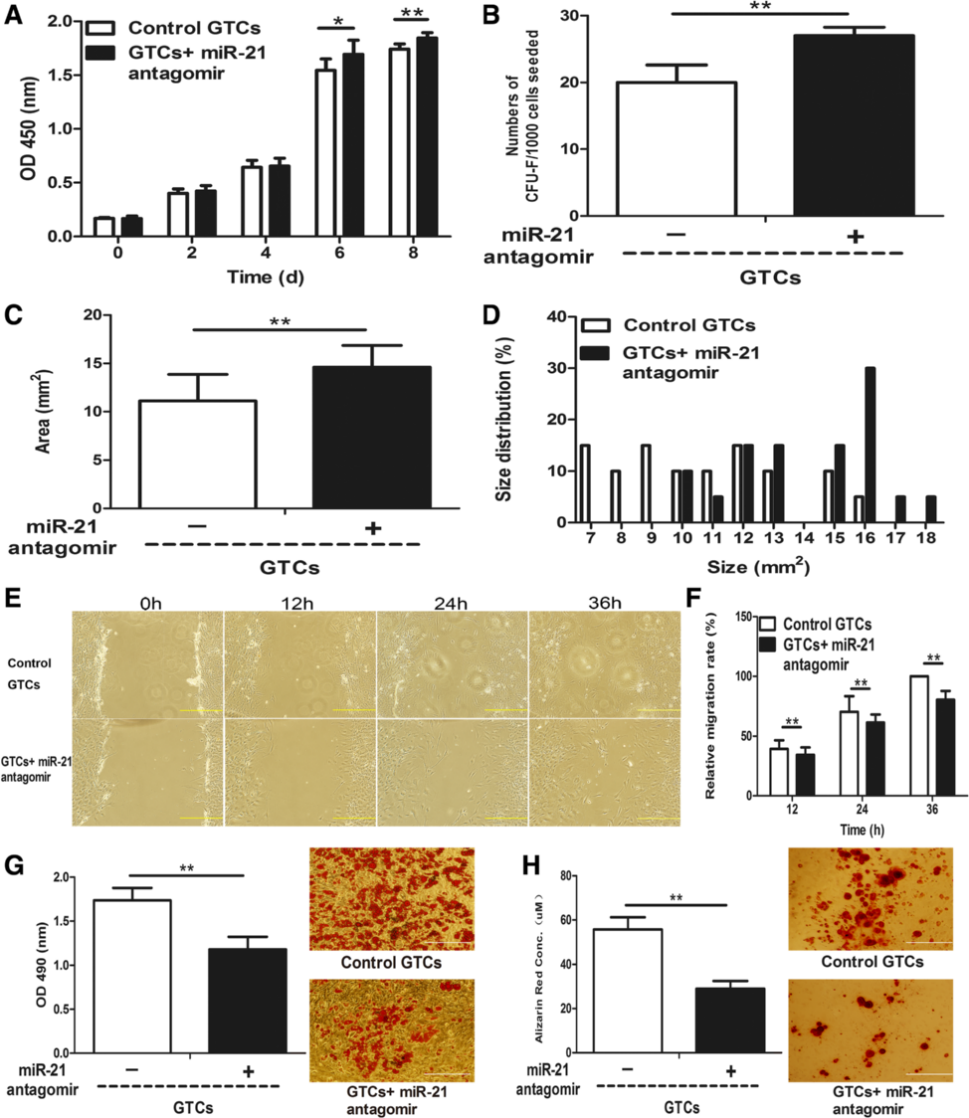
miR-21 negatively regulates stemness-related properties of granulation tissue-derived cells. Passage 2 granulation tissue-derived cells (GTCs) were pretreated with microRNA (miR)-21 antagomir (50 nM) or control antagomir (50 nM) for 48 hours. **(A)** The proliferation ability was detected by CCK-8 every 2 days after seeding. **(B)** Colony-forming assay of GTCs pretreated with miR-21 antagomir and control antagomir. Colonies presented as mean ± standard deviation (*n* = 6 wells per group). Twenty colonies were randomly chosen in each group for analyzing **(C)** average colony sizes and **(D)** size distribution. **(E)** Representative migration photographs of GTCs pretreated with miR-21 antagomir and control antagomir at indicated time points. **(F)** Relative migration rate presented as mean ± standard deviation (*n* = 6 per time point for each group). **(G)** Adipogenic and **(H)** osteogenic differentiation of GTCs pretreated with miR-21 antagomir or control antagomir were measured by staining with Oil-Red O and Alizarin Red and quantifying them respectively. CFU, colony-forming units; d, days; h, hours; OD, optical density. *P < 0.05, **P < 0.01. Scale bar = 500 μm.

Our recent study has shown that miR-21 was upregulated in granulation tissues after wounding by *in situ* hybridization [11]. Here, we detected the miR-21 level in GTCs and found it was significantly (29.8-fold change) increased compared with nonwounded dermal cells (Figure 4A). Thus, we further studied the roles of miR-21 in the stemness-related properties of GTCs by antagonizing miR-21 expression with miR-21 antagomir. Surprisingly, the miR-21 antagomir increased the proliferation ability of GTCs (Figure 5A). Further, the colony-forming unit fibroblast assay showed that miR-21 antagomir-treated GTCs formed more colonies (27 ± 1.26) compared with GTCs with control antagomir (20 ± 2.61; Figure 5B), and the sizes of the colonies were also larger in miR-21 antagomir-treated GTCs (14.61 ± 2.25) compared with GTCs with control antagomir (11.11 ± 2.75; Figure 5C,D). However, miR-21 antagomir reduced migration of GTCs in a scratch wound closure assay (Figure 5E,F), and the adipogenic and osteogenic differentiation were also decreased in miR-21 antagomir-treated GTCs (Figure 5G,H). These results suggested that blockade of miR-21 could decrease the migration and multidifferentiation of GTCs, but promote their proliferation and self-renewing capabilities.

**Figure 6:**
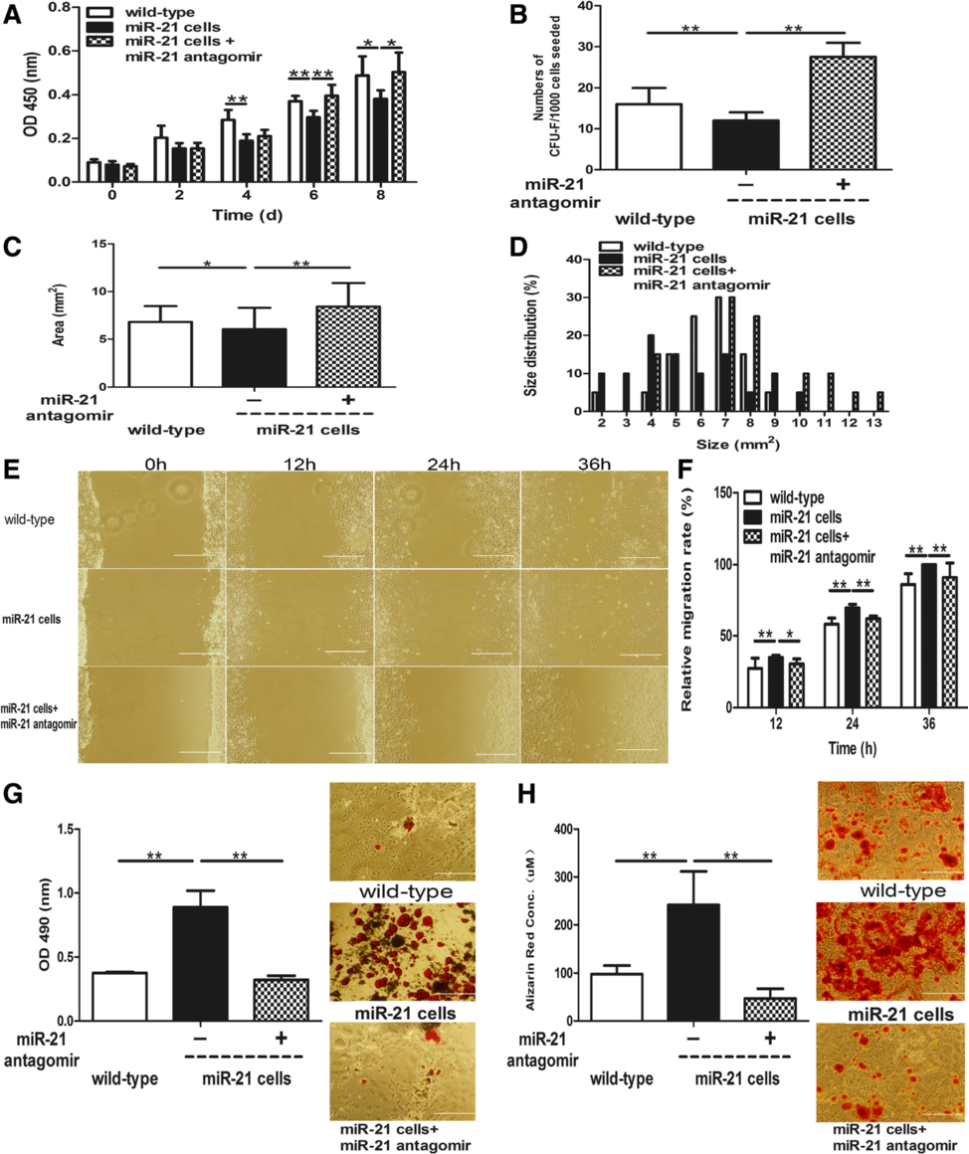
miR-21 negatively regulates stemness-related properties of miR-21 knock-in neonatal dermal cells. Passage 2 microRNA (miR)-21 knock-in neonatal dermal cells were pretreated with miR-21 antagomir or control antagomir (50 nM) for 48 hours, and wild-type neonatal dermal cells were treated with control antagomir (50 nM) for 48 hours. **(A)** The proliferation ability of the three groups was detected every 2 days after seeding. Colony formation assay including **(B)** colony numbers, **(C)** colony sizes, and **(D)** colony size distribution of the three groups was also measured. **(E)** Representative photographs of migration of the three groups at indicated time points. **(F)** Relative migration rate of the three groups presented as mean ± standard deviation (*n* = 6 per time point for each group). **(G)** Adipogenic and **(H)** osteogenic differentiation of the three groups were measured by staining with Oil-Red O and Alizarin Red and quantifying them respectively. CFU, colony-forming units; d, days; h, hours; miR-21 cells, miR-21 knock-in neonatal dermal cells; wild-type, wild-type neonatal dermal cells; OD, optical density. *P < 0.05, **P < 0.01. Scale bar = 500 μm.

To further confirm whether miR-21 negatively regulates stemness-related properties of dermal derived cells, we verified its effects on a miR-21 knock-in neonatal dermal cell model. miR-21 knock-in neonatal dermal cells showed decreased proliferation compared with wild-type neonatal dermal cells (Figure 6A). In addition, they formed less colonies (12 ± 2.0) compared with wild-type neonatal dermal cells (16.0 ± 3.97; Figure 6B). Moreover, the sizes of the colonies of miR-21 knock-in neonatal dermal cells were also smaller (Figure 6C,D). Further, miR-21 antagomir could significantly reverse the decrease of the proliferation and colony-forming abilities (Figure 6A,B,C,D). However, miR-21 knock-in neonatal dermal cells showed increased migration (Figure 6E,F) and adipogenic and osteogenic differentiation (Figure 6G,H) compared with wild-type neonatal dermal cells, and miR-21 antagomir could reverse that increase (Figure 6E,F,G,H). These results further confirmed that miR-21 could decrease proliferation and self-renewing capabilities, but could promote migration and multidifferentiation of dermal-derived cells.

### miR-21 negatively regulates the stemness-related properties of dermal-derived cells via a ROS-dependent pathway

Previous studies have shown that ROS were upregulated at the wound site [21,22], but their functions in wound healing are not fully identified. We measured the ROS level in GTCs, and found it was significantly increased in GTCs (261.55 ± 19.26) compared with nonwounded dermal cells (100.00 ± 6.88; Figure 4B). To determine the relationship between miR-21 and ROS, we antagonized miR-21 expression in GTCs and found that the ROS level decreased significantly (115.14 ± 7.91; Figure 4B). On the other hand, we scavenged intracellular ROS in GTCs with a free radical scavenger (NAC), and the miR-21 expression did not change significantly compared with control GTCs (<0.5-fold change; Figure 4A). We next verified the effect of miR-21 on ROS expression in the miR-21 knock-in neonatal dermal cell model. As shown in Figure 4D, the ROS level in miR-21 knock-in neonatal dermal cells (122.96 ± 12.9) was increased compared with wild-type cells (100 ± 2.12), and miR-21 antagomir reversed the increase. In addition, NAC also did not affect miR-21 expression in miR-21 knock-in neonatal dermal cells (<0.5-fold change; Figure 4C).

**Figure 7:**
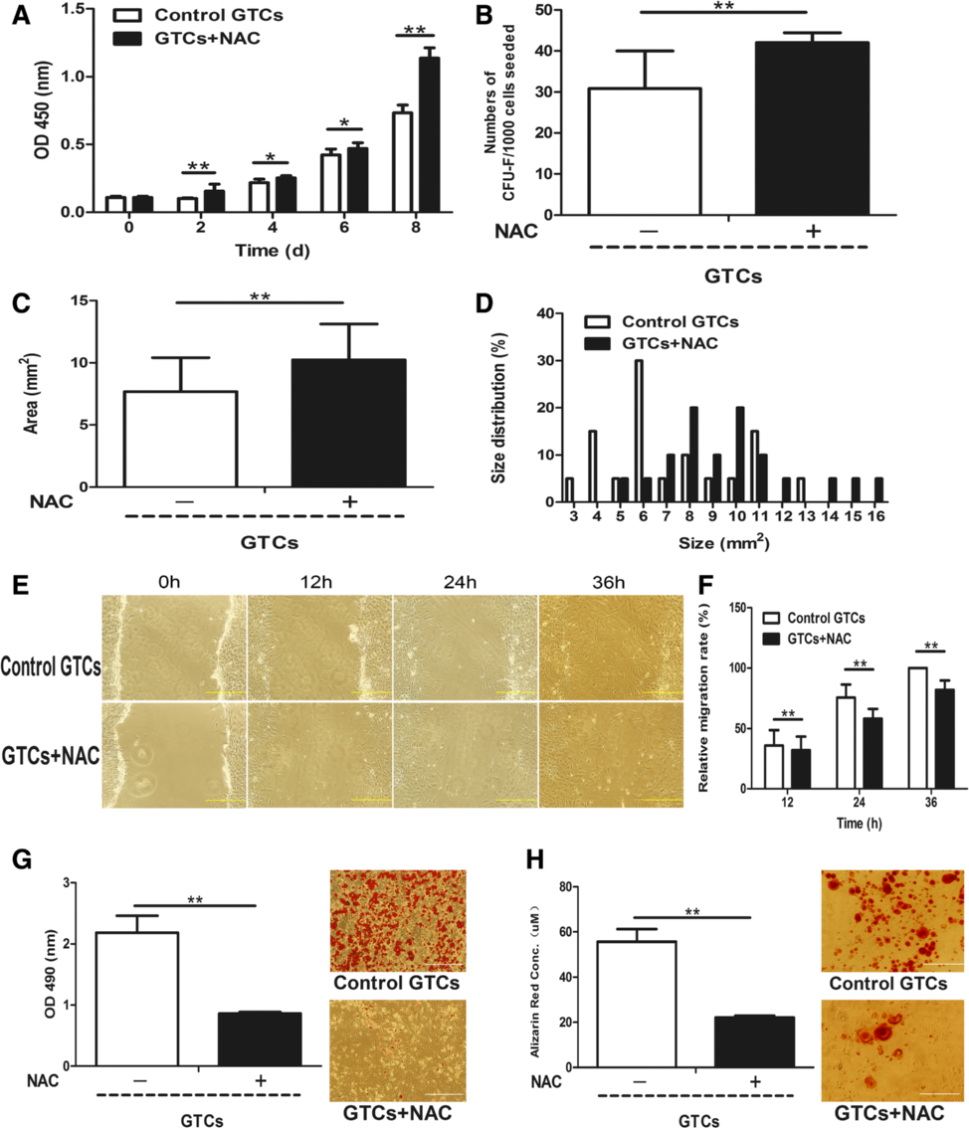
miR-21 negatively regulates stemness-related properties of granulation tissue-derived cells via a reactive oxygen species-dependent pathway. *N*-acetyl cysteine (NAC; 5 mM) was added into culture medium and adipogenic and osteogenic induced medium of granulation tissue-derived cells (GTCs) to scavenge the intracellular reactive oxygen species (ROS). **(A)** Proliferation of GTCs with and without NAC was measured every 2 days after seeding. Colony formation assay including **(B)** colony numbers, **(C)** average colony sizes and **(D)** colony size distribution of GTCs with and without NAC was present. **(E)** Representative migration photographs of GTCs with and without NAC at indicated time points. **(F)** The migration rate of the two groups presented as mean ± standard deviation (*n* = 6 per time point for each group). **(G)** The quantification and representative photographs of the Oil-Red O staining of the adipogenic differentiation of GTCs with and without NAC. **(H)** Quantification and representative photographs of the Alizarin Red staining of the osteogenic differentiation of GTCs and GTCs with NAC. CFU, colony-forming units; d, days; h, hours, OD, optical density. *P <0.05; **P <0.01. Scale bar = 500 μm.

**Figure 8:**
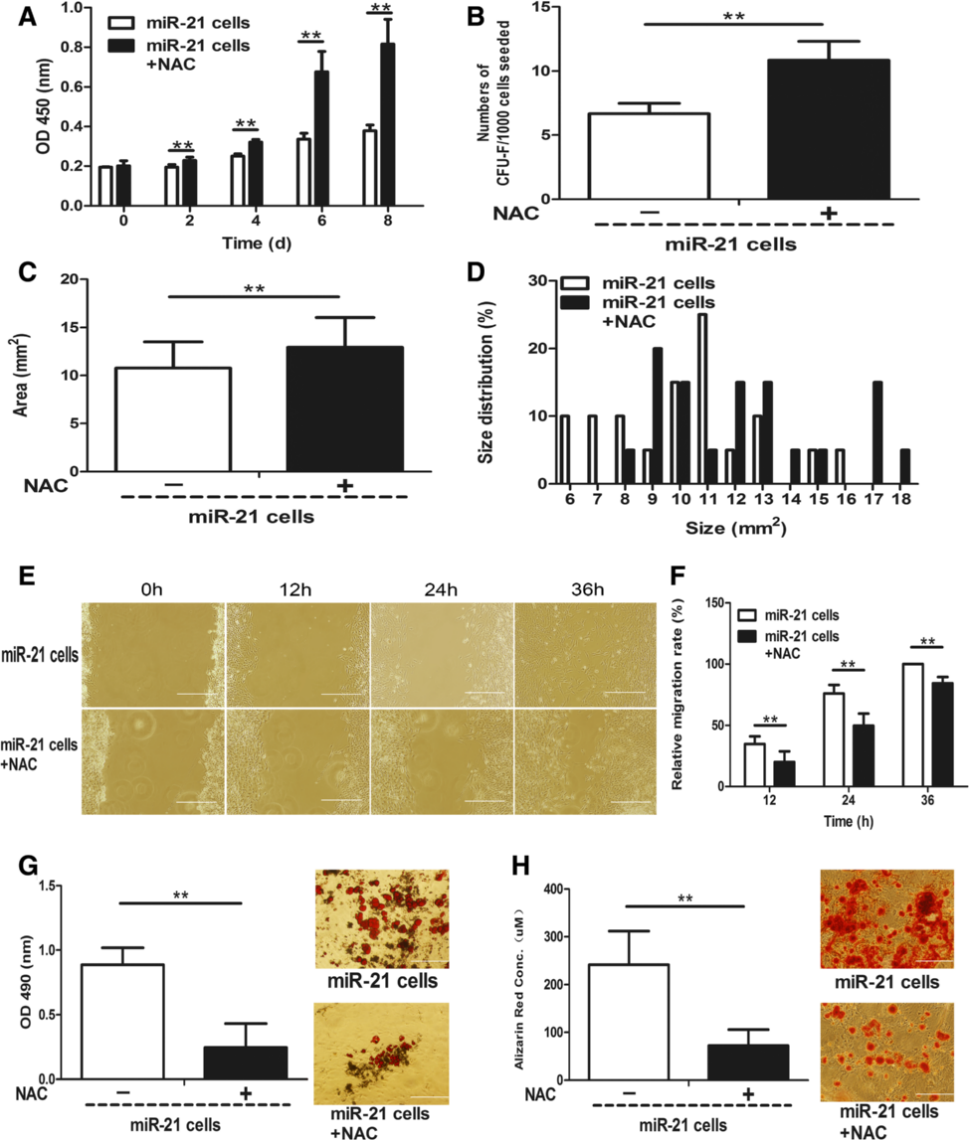
miR-21 negatively regulates stemness-related properties of miR-21 knock-in neonatal dermal cells via a reactive oxygen species dependent pathway. *N*-acetyl cysteine (NAC; 5 mM) was added into culture medium and adipogenic and osteogenic induced medium of miR-21 knock-in neonatal dermal cells to scavenge intracellular reactive oxygen species (ROS), and stemness related-properties of miR-21 knock-in neonatal dermal cells with and without NAC were detected. **(A)** Proliferation was measured every 2 days after seeding. Colony formation assay including **(B)** colony numbers, **(C)** average colony sizes, and **(D)** colony size distribution was present. **(E)** Representative migration photographs at indicated time points. **(F)** Relative migration rate presented as mean ± standard deviation (*n* = 6 per time point for each group). **(G)** Quantification and representative photographs of the Oil-Red O staining of the adipogenic differentiation. **(H)** Quantification and representative photographs of the Alizarin Red staining of the osteogenic differentiation. CFU, colony-forming units; d, days; h, hours; miR-21 cells, miR-21 knock in neonatal dermal cells; OD, optical density. **P <0.01. Scale bar = 500 μm.

Further, to determine whether miR-21 regulates stemness-related properties of dermal-derived cells through a ROS-dependent pathway, we scavenged endogenous ROS in the two miR-21 up-regulated cell populations – GTCs and miR-21 knock-in neonatal dermal cells with NAC – and tested their stemness-related properties. As shown in Figure 7A, NAC-treated GTCs exhibited increased proliferation ability compared to control GTCs. In addition, the formed colonies (42 ± 2.45; Figure 7B) and colony sizes (10.24 ± 2.89; Figure 7C, D) of NAC-treated GTCs were increased compared with control GTCs (30.83 ± 9.15 and 7.68 ± 2.73, respectively). However, the migration (Figure 7E,F) and adipogenic (Figure 7G) and osteogenic (Figure 7H) differentiation of NAC-treated GTCs were decreased compared with control GTCs. Moreover, NAC-treated miR-21 knock-in neonatal dermal cells also showed increased proliferation (Figure 8A) and formed more (Figure 8B) and larger colonies (Figure 8C,D) compared with control miR-21 knock-in neonatal dermal cells. Consistent with GTCs, the migration (Figure 8E,F) and adipogenic (Figure 8G) and osteogenic (Figure 8H) differentiation of NAC-treated miR-21 knock-in neonatal dermal cells were also decreased compared with control miR-21 knock-in neonatal dermal cells. All the above data suggest that miR-21 could regulate stemness-related properties of dermal derived cells at least partially via a ROS-dependent pathway.

## Discussion

An ideal stem cell source should be easily accessible, immunologically compatible, capable to rapidly expand in culture, and amenable to stable differentiation or transdifferentiation. Skin, the largest organ of the body with easy accessibility, is a promising reservoir for adult stem cells. In the epidermis, epidermal stem cells [23], hair follicle stem cells, and melanocyte stem cells [6] are resident. Recently, dermis has been proven to contain stem cell populations [24-26]. However, the stem cell proportion in the adult was relatively rare. Previous studies demonstrated that the dermal stem cells decreased in number and capabilities of self-renewing or proliferation with aging [8,27]. Skin frequently suffers from injury, and the resident stem cells are proposed to be activated for proliferation and differentiation by various factors from the wound microenvironment including cytokines, growth factors, and extracellular matrix components released by platelets, leukocytes, and macrophages to fulfill the defects [28]. In this study, we showed that dermal stem/progenitor cells were quiescent in nonwounded skin and were activated after wounding and enriched in the granulation tissue. The isolated GTCs exhibited enhanced proliferation, self-renewing, and multidifferentiation capabilities compared with nonwounded dermal cells. Very recently, studies have explored the potential of granulation tissue-derived stem cells in the tissue repair of liver and kidney [9,10], which further suggests that granulation tissue is a promising source of adult stem cells.

miRNAs are evaluated to regulate the expression of one-third of genes, and their roles in wound healing have already attracted much attention [29-32]. In a recent study, we performed miRNA microarray profiling to estimate the changes of the miRNAs at the stage of granulation formation during wound healing, and found that the expression of at least 54 miRNAs changed significantly [11]. miR-21 was one of the most significantly upregulated miRNAs after wounding, and the increased expression of miR-21 at the wound sites was proposed to be modulated by inflammatory factors especially transforming growth factor beta [33,34]. Previous studies showed that the upregulated miR-21 affected multiple aspects of the healing process by booting re-epithelialization [32], promoting migration of fibroblasts to the wound site [35], and enhancing collagen deposition [11,36]. In this study, we continued to explore the roles of miR-21 in GTCs. Data showed that miR-21 was upregulated in GTCs. Blockade of miR-21 promoted proliferation and self-renewing of GTCs, but decreased their migration and differentiation, which presented as a negative modulator of the stemness-related properties. Previous studies demonstrated that miR-21 showed similar effects in stromal cells. It has been identified that miR-21 could decrease the proliferation of adipose tissue-derived mesenchymal stem cells [37], but promote adipogenic differentiation [38]. In condition with hypoxia and serum deprivation, overexpression of miR-21 inhibited apoptosis of mesenchymal stem cells [39]. However, miR-21 showed different effects in epithelial cells. In a liver regeneration model, miR-21 promoted hepatocyte proliferation by facilitating rapid cyclin D_1_ translation [40]. Another study showed that miR-21 promoted the senescence of endothelial progenitors by suppressing Hmga2 expression [41].

It is well established that ROS play important roles in regulating cellular functions such as cell proliferation, migration, differentiation, apoptosis, and death [42-46]. According to previous studies, the ROS level at the wound site was upregulated at the early stage of wound healing [22]. In this study, the endogenous ROS level in GTCs was also increased. Previously, ROS was shown to upregulate miR-21expression via different pathways in different cell types [47-53]. Interestingly, our data showed that scavenging endogenous ROS did not decrease miR-21 expression. On the contrary, inhibiting miR-21 expression suppressed the ROS level significantly. In other studies, miR-21 was also shown to regulate the endogenous ROS level. In the process of kidney fibrogenesis, miR-21 upregulates ROS level by repressing the mitochondrial inhibitor of ROS generation Mpv17l, and enhances oxidative kidney damage [54]. miR-21 also targets superoxide dismutase 3 and tumor necrosis factor alpha, and promotes tumorigenesis to a larger extent by regulating endogenous ROS level [55]. Furthermore, we explored that scavenging endogenous ROS could mimic the effects of miR-21 antagomir on GTC properties, which suggested that miR-21 regulated their stemness-related properties at least partially via a ROS-dependent pathway.

## Conclusions

The cell source is a keystone for stem cell-based therapy. Previous studies have demonstrated that skin dermis is an abundant stem cell source and is easily accessible. However, stem cell numbers and plasticity deplete with aging. This study demonstrated that dermal stem/progenitor cells were activated upon injury and enriched in granulation tissue with enhanced stemness-related properties and therapeutic potential, and the miR-21/ROS pathway negatively regulate stemness-related properties of GTCs. Our results suggest that skin granulation tissue represents a promising stem cell source for tissue repair and regeneration.

## Additional file

Additional file 1: Figure S1. Showing transient proliferation of skin dermis after wounding. BrdU (100 mg/kg) was injected intraperitoneally 2 hours before the nonwounded and wound tissues sampling. Normal skin tissues or wounded tissues (*n* = 3 per time point) at 1, 3, 5, 7, 9, 12, 15, and 20 days post wounding were harvested and fixed 48 hours in 4% paraformaldehyde and embedded in paraffin. Sections of 4 mm were immunostained with BrdU antibody and color developed by diaminobenzidine, and counterstained with hematoxylin. d, days after wounding. Scale bar = 100 μm.

## Abbreviations

BrdU: 5-bromo-2′-deoxyuridine
GTC: granulation tissue-derived cell
LRC: label-retaining cell
miR: microRNA
NAC: *N*-acetylcysteine
PBS: phosphate-buffered saline
ROS: reactive oxygen species
TMMU: Third Military Medicine University

## References

[1] Watt FM (1998). Epidermal stem cells: markers, patterning and the control of stem cell fate. Philos Trans R Soc Lond B Biol Sci.

[2] Hughes S (2002). Epidermal stem cells. J Pathol.

[3] Ghazizadeh S, Taichman LB (2001). Multiple classes of stem cells in cutaneous epithelium: a lineage analysis of adult mouse skin. EMBO J.

[4] Uzarska M, Porowinska D, Bajek A, Drewa T (2013). Epidermal stem cells – biology and potential applications in regenerative medicine. Postepy Biochem.

[5] Myung P, Andl T, Ito M (2009). Defining the hair follicle stem cell (Part II). J Cutan Pathol.

[6] Gola M, Czajkowski R, Bajek A, Dura A, Drewa T (2012). Melanocyte stem cells: biology and current aspects. Med Sci Monit.

[7] Chen Z, Wang Y, Shi C. Therapeutic implications of newly identified stem cell populations from the skin dermis. Cell Transplant. 2014. doi:10.3727/096368914x682431. 10.3727/096368914X682431 24972091

[8] Gago N, Perez-Lopez V, Sanz-Jaka JP, Cormenzana P, Eizaguirre I, Bernad A (2009). Age-dependent depletion of human skin-derived progenitor cells. Stem Cells.

[9] Patel J, Gudehithlu KP, Dunea G, Arruda JA, Singh AK (2010). Foreign body-induced granulation tissue is a source of adult stem cells. Transl Res.

[10] Patel J, Pancholi N, Gudehithlu KP, Sethupathi P, Hart PD, Dunea G (2012). Stem cells from foreign body granulation tissue accelerate recovery from acute kidney injury. Nephrol Dial Transplant.

[11] Wang T, Feng Y, Sun H, Zhang L, Hao L, Shi C (2012). miR-21 regulates skin wound healing by targeting multiple aspects of the healing process. Am J Pathol.

[12] Xue Z, Yan H, Li J, Liang S, Cai X, Chen X (2012). Identification of cancer stem cells in vincristine preconditioned SGC7901 gastric cancer cell line. J Cell Biochem.

[13] Chen C, Ridzon DA, Broomer AJ, Zhou Z, Lee DH, Nguyen JT (2005). Real-time quantification of microRNAs by stem-loop RT-PCR. Nucleic Acids Res.

[14] Zhang H, Zhai Z, Wang Y, Zhang J, Wu H, Li C (2012). Resveratrol ameliorates ionizing irradiation-induced long-term hematopoietic stem cell injury in mice. Free Radic Biol Med.

[15] Saleh FA, Whyte M, Ashton P, Genever PG (2011). Regulation of mesenchymal stem cell activity by endothelial cells. Stem Cells Dev.

[16] Liang CC, Park AY, Guan JL (2007). In vitro scratch assay: a convenient and inexpensive method for analysis of cell migration in vitro. Nat Protoc.

[17] Morris RJ, Potten CS (1994). Slowly cycling (label-retaining) epidermal cells behave like clonogenic stem cells in vitro. Cell Prolif.

[18] Li S, Sun G, Murai K, Ye P, Shi Y (2012). Characterization of TLX expression in neural stem cells and progenitor cells in adult brains. PLoS One.

[19] Kurzrock EA, Lieu DK, Degraffenried LA, Chan CW, Isseroff RR (2008). Label-retaining cells of the bladder: candidate urothelial stem cells. Am J Physiol Renal Physiol.

[20] Haubner F, Ohmann E, Pohl F, Strutz J, Gassner HG (2012). Wound healing after radiation therapy: review of the literature. Radiat Oncol.

[21] Hanselmann C, Mauch C, Werner S (2001). Haem oxygenase-1: a novel player in cutaneous wound repair and psoriasis?. Biochem J.

[22] Schafer M, Werner S (2008). Oxidative stress in normal and impaired wound repair. Pharmacol Res.

[23] Plikus MV, Gay DL, Treffeisen E, Wang A, Supapannachart RJ, Cotsarelis G (2012). Epithelial stem cells and implications for wound repair. Semin Cell Dev Biol.

[24] Shi CM, Chen TM (2001). Isolation and culture of multipotent stem cells derived from neonatal rat dermis. Acta Academiae Medicinae Militaris Tertiae.

[25] Toma JG, Akhavan M, Fernandes KJ, Barnabe-Heider F, Sadikot A, Kaplan DR (2001). Isolation of multipotent adult stem cells from the dermis of mammalian skin. Nat Cell Biol.

[26] Young HE, Steele TA, Bray RA, Hudson J, Floyd JA, Hawkins K (2001). Human reserve pluripotent mesenchymal stem cells are present in the connective tissues of skeletal muscle and dermis derived from fetal, adult, and geriatric donors. Anat Rec.

[27] Liu S, Wang X, Zhou J, Cao Y, Wang F, Duan E (2011). The PI3K–Akt pathway inhibits senescence and promotes self-renewal of human skin-derived precursors in vitro. Aging Cell.

[28] Werner S, Grose R (2003). Regulation of wound healing by growth factors and cytokines. Physiol Rev.

[29] Biswas S, Roy S, Banerjee J, Hussain SR, Khanna S, Meenakshisundaram G (2010). Hypoxia inducible microRNA 210 attenuates keratinocyte proliferation and impairs closure in a murine model of ischemic wounds. Proc Natl Acad Sci U S A.

[30] Wang T, Zhang L, Shi C, Sun H, Wang J, Li R (2012). TGF-beta-induced miR-21 negatively regulates the antiproliferative activity but has no effect on EMT of TGF-beta in HaCaT cells. Int J Biochem Cell Biol.

[31] Bertero T, Gastaldi C, Bourget-Ponzio I, Imbert V, Loubat A, Selva E (2011). miR-483-3p controls proliferation in wounded epithelial cells. FASEB J.

[32] Yang X, Wang J, Guo SL, Fan KJ, Li J, Wang YL (2011). miR-21 promotes keratinocyte migration and re-epithelialization during wound healing. Int J Biol Sci.

[33] Dey N, Ghosh-Choudhury N, Kasinath BS, Choudhury GG (2012). TGFbeta-stimulated microRNA-21 utilizes PTEN to orchestrate AKT/mTORC1 signaling for mesangial cell hypertrophy and matrix expansion. PLoS One.

[34] Zhu H, Luo H, Li Y, Zhou Y, Jiang Y, Chai J (2013). MicroRNA-21 in scleroderma fibrosis and its function in TGF-beta-regulated fibrosis-related genes expression. J Clin Immunol.

[35] Madhyastha R, Madhyastha H, Nakajima Y, Omura S, Maruyama M (2012). MicroRNA signature in diabetic wound healing: promotive role of miR-21 in fibroblast migration. Int Wound J.

[36] Liu G, Friggeri A, Yang Y, Milosevic J, Ding Q, Thannickal VJ (2010). miR-21 mediates fibrogenic activation of pulmonary fibroblasts and lung fibrosis. J Exp Med.

[37] Kim YJ, Hwang SH, Cho HH, Shin KK, Bae YC, Jung JS (2012). MicroRNA 21 regulates the proliferation of human adipose tissue-derived mesenchymal stem cells and high-fat diet-induced obesity alters microRNA 21 expression in white adipose tissues. J Cell Physiol.

[38] Kim YJ, Hwang SJ, Bae YC, Jung JS (2009). MiR-21 regulates adipogenic differentiation through the modulation of TGF-beta signaling in mesenchymal stem cells derived from human adipose tissue. Stem Cells.

[39] Nie Y, Han BM, Liu XB, Yang JJ, Wang F, Cong XF (2011). Identification of MicroRNAs involved in hypoxia- and serum deprivation-induced apoptosis in mesenchymal stem cells. Int J Biol Sci.

[40] Ng R, Song G, Roll GR, Frandsen NM, Willenbring H (2012). A microRNA-21 surge facilitates rapid cyclin D1 translation and cell cycle progression in mouse liver regeneration. J Clin Invest.

[41] Zhu S, Deng S, Ma Q, Zhang T, Jia C, Zhuo D (2013). MicroRNA-10A* and MicroRNA-21 modulate endothelial progenitor cell senescence via suppressing high-mobility group A2. Circ Res.

[42] Irani K (2000). Oxidant signaling in vascular cell growth, death, and survival: a review of the roles of reactive oxygen species in smooth muscle and endothelial cell mitogenic and apoptotic signaling. Circ Res.

[43] Vandenbroucke K, Robbens S, Vandepoele K, Inze D, Van de Peer Y, Van Breusegem F (2008). Hydrogen peroxide-induced gene expression across kingdoms: a comparative analysis. Mol Biol Evol.

[44] Lu FJ, Tseng TH, Lee WJ, Yen CC, Yin YF, Liao CW (2006). Promoting neoplastic transformation of humic acid in mouse epidermal JB6 Cl41 cells. Chem Biol Interact.

[45] De Felice B, Garbi C, Santoriello M, Santillo A, Wilson RR (2009). Differential apoptosis markers in human keloids and hypertrophic scars fibroblasts. Mol Cell Biochem.

[46] Scharstuhl A, Mutsaers HA, Pennings SW, Szarek WA, Russel FG, Wagener FA (2009). Curcumin-induced fibroblast apoptosis and in vitro wound contraction are regulated by antioxidants and heme oxygenase: implications for scar formation. J Cell Mol Med.

[47] Cheng Y, Liu X, Zhang S, Lin Y, Yang J, Zhang C (2009). MicroRNA-21 protects against the H(2)O(2)-induced injury on cardiac myocytes via its target gene PDCD4. J Mol Cell Cardiol.

[48] Lin Y, Liu X, Cheng Y, Yang J, Huo Y, Zhang C (2009). Involvement of MicroRNAs in hydrogen peroxide-mediated gene regulation and cellular injury response in vascular smooth muscle cells. J Biol Chem.

[49] Wang X, Cheng Y, Liu X, Yang J, Munoz D, Zhang C (2011). Unexpected pro-injury effect of propofol on vascular smooth muscle cells with increased oxidative stress. Crit Care Med.

[50] Wei C, Li L, Kim IK, Sun P, Gupta S (2014). NF-kappaB mediated miR-21 regulation in cardiomyocytes apoptosis under oxidative stress. Free Radic Res.

[51] Ling M, Li Y, Xu Y, Pang Y, Shen L, Jiang R (2012). Regulation of miRNA-21 by reactive oxygen species-activated ERK/NF-kappaB in arsenite-induced cell transformation. Free Radic Biol Med.

[52] Jajoo S, Mukherjea D, Kaur T, Sheehan KE, Sheth S, Borse V (2013). Essential role of NADPH oxidase-dependent reactive oxygen species generation in regulating microRNA-21 expression and function in prostate cancer. Antioxid Redox Signal.

[53] Saxena A, Shoeb M, Ramana KV, Srivastava SK (2013). Aldose reductase inhibition suppresses colon cancer cell viability by modulating microRNA-21 mediated programmed cell death 4 (PDCD4) expression. Eur J Cancer.

[54] Chau BN, Xin C, Hartner J, Ren S, Castano AP, Linn G (2012). MicroRNA-21 promotes fibrosis of the kidney by silencing metabolic pathways. Sci Transl Med.

[55] Zhang X, Ng WL, Wang P, Tian L, Werner E, Wang H (2012). MicroRNA-21 modulates the levels of reactive oxygen species by targeting SOD3 and TNFalpha. Cancer Res.

