# Supplementary figures and images for: Activation and regulation of the granulation tissue derived cells with stemness-related properties
Source: Stem Cell Res Ther. 2015 Apr 29;6(1):85. doi: 10.1186/s13287-015-0070-9 (PMC4446126; doi:10.1186/s13287-015-0070-9)

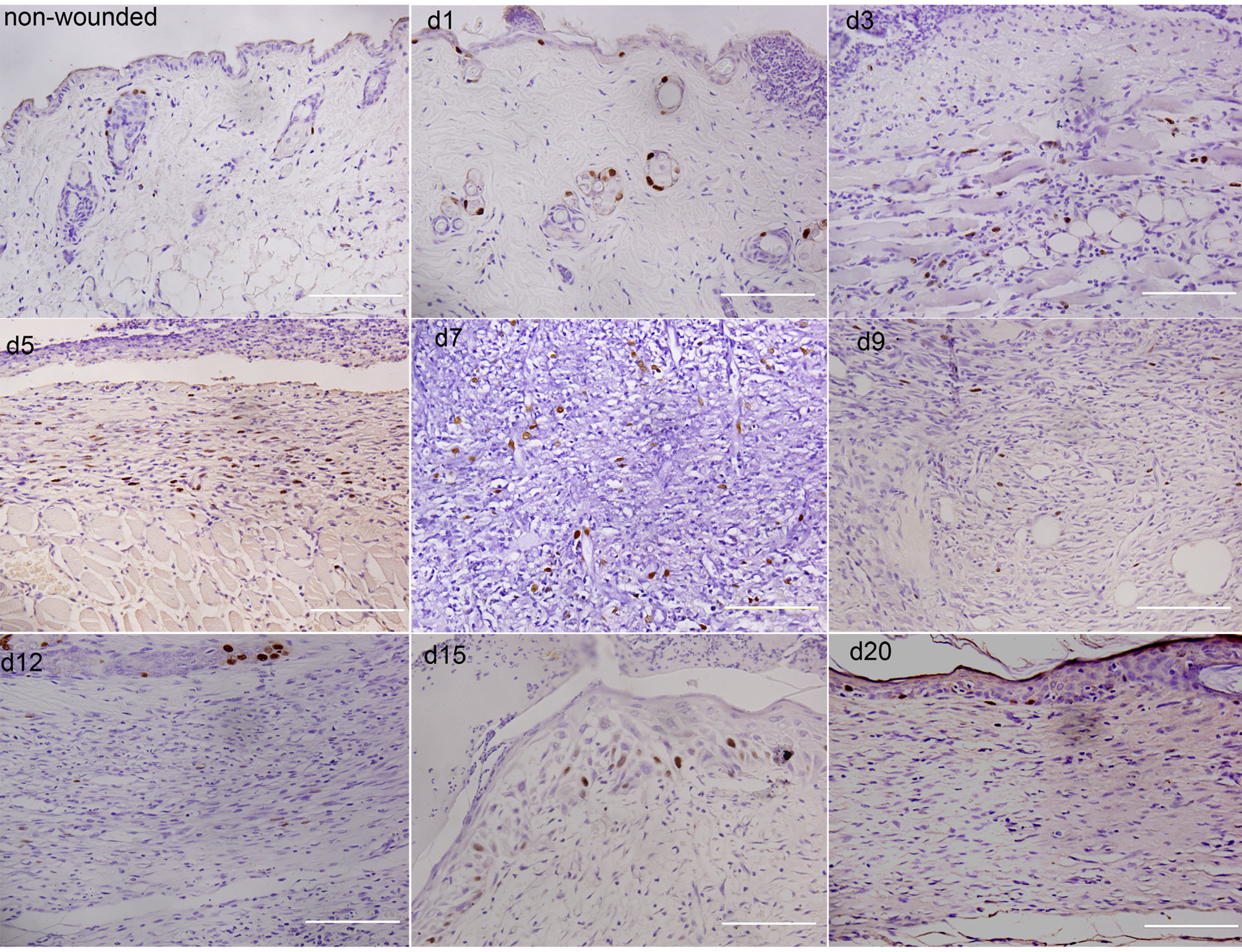

Supplement: Additional file 1: Figure S1. — Showing transient proliferation of skin dermis after wounding. BrdU (100 mg/kg) was injected intraperitoneally 2 hours before the nonwounded and wound tissues sampling. Normal skin tissues or wounded tissues (n = 3 per time point) at 1, 3, 5, 7, 9, 12, 15, and 20 days post wounding were harvested and fixed 48 hours in 4% paraformaldehyde and embedded in paraffin. Sections of 4 mm were immunostained with BrdU antibody and color developed by diaminobenzidine, and counterstained with hematoxylin. d, days after wounding. Scale bar = 100 μm. [file 13287_2015_70_MOESM1_ESM.tiff]
